# Gastrointestinal stromal tumors regulate macrophage M2 polarization through the MIF/CXCR4 axis to immune escape

**DOI:** 10.3389/fimmu.2024.1431535

**Published:** 2024-10-11

**Authors:** Shuo-meng Xiao, Rui Xu, Ying-xin Yang, Rui Zhao, Yuan Xie, Xu-dan Lei, Xiao-ting Wu

**Affiliations:** ^1^ Department of Gastric Surgery, Sichuan Clinical Research Center for Cancer, Sichuan Cancer Hospital and Institute, Sichuan Cancer Center, Affiliated Cancer Hospital of University of Electronic Science and Technology of China, Chengdu, Sichuan, China; ^2^ Department of Oncology, The First People’s Hospital of Dali, Dali City, Yunnan, China; ^3^ Department of Gastrointestinal Surgery, West China School of Medicine and West China Hospital, Sichuan University, Chengdu, Sichuan, China; ^4^ Radiation Oncology Key Laboratory of Sichuan Province, Department of Experimental Research, Sichuan Cancer Hospital and Institute, Sichuan Cancer Center, Affiliated Cancer Hospital of University of Electronic Science and Technology of China, Chengdu, Sichuan, China

**Keywords:** gastrointestinal stromal tumor, macrophage, single-cell RNA sequencing, tumor microenvironment, M2 polarization

## Abstract

**Purpose:**

The infiltration of immune cells and their roles of the infiltrating-immune cells in gastrointestinal stromal tumor (GIST) is still unclear. We aimed to discover the infiltration cell types and the relationship between the infiltrating-immune cells and the progression of GIST.

**Experimental design:**

Single-cell RNA sequencing were performed to discover types of the infiltrating-immune cells and to analyze CellChat between cells. Immunohistochemistry of 80 GIST samples were used to clarify the relation between macrophages and recurrence risk. *In vitro*, flow cytometry and Real-time PCR were performed to uncover a potential mechanism of tumor cell regulation of macrophages.

**Results:**

Tumor cells, macrophages, and T-cells were the predominant cell types. The MIF/CXCR4 axis was the most common ligand–receptor interaction between macrophages and tumor cells. As the risk increased, expression levels of CD68, CD206, MIF, and CXCR4 gradually increased. *In vitro*, we found that GIST882 was able to secrete MIF and GIST882 cell supernatant upregulated M2 polarization. Real-time PCR showed that expression levels of IL-10 mRNA and Arginase-1 mRNA were also the highest in the GIST882 cell supernatant group.

**Conclusions:**

These findings identify that macrophages are the most abundant infiltrating cells in GIST. The MIF/CXCR4 axis is the most common ligand–receptor interaction between macrophages and tumor cells. GIST cells can regulate macrophage M2 polarization through the MIF/CXCR4 axis.

## Introduction

Gastrointestinal stromal tumor (GIST) is the most common mesenchymal tumors in the gastrointestinal tract (1). It is well-known that GISTs originate from the interstitial cells of Cajal and that GISTs are characterized by acquired gene mutations. Specifically, *KIT* (75–80%) and *PDGFRA* (5–10%) are the most common gain-of-function mutations (2, 3).

The risk stratification based on tumor size, tumor site, and mitotic count is often used to predict the recurrence risk. Targeted therapy is the first choice for the patients with metastatic disease and those with intermediate or high recurrence risk (4). Imatinib, from the first generation of tyrosine kinase inhibitors (TKIs), is the most effective drug. However, patients invariably develop drug resistance. The second generation and the third generation of TKIs have limited benefit and more drug side effects (5). Thus, it is important to develop new treatment strategies.

Recently, the monoclonal antibodies against immunological checkpoints PD-1 and CTLA-4 have been used in most tumors to improve overall survival (6–8). However, not all the patients can benefit from the immune therapy. Factors such as PD-1/PD-L1, tumor mutational burden, and microsatellite instability have been suggested to predict treatment response. Tumor-infiltrating immune cells seem to play an important role in treatment responses and tumor progression (9–11). Available studies have shown abundant immune cell infiltration in GIST (5, 12–14). Moreover, it has been demonstrated that imatinib could lead to the activation of CD8^+^ T cells and that CD8^+^ T cells also promote imatinib’s antitumor effects (5, 15). However, CD8^+^ T cells become exhausted and macrophage count increases with tumor progression (12, 16). Although abundant immune cell infiltration has been found in GIST microenvironment, clinical studies have shown that immunotherapy is not ideal (17, 18). Thus, it is important to clarify the roles of infiltrated immune cells in the progression of GIST.

In this study, we aimed to determine the role of macrophages during tumor progression and cell-chat types between macrophages and tumor cells.

## Patients and methods

### Single-cell RNA sequencing

Two patients (G01 and G02) were recruited for single-cell transcriptome analysis. The two tumors were both located on the greater curvature of the gastric body. The patient G01 underwent surgical resection, and the patient G02 underwent endoscopic resection. Immunohistochemical results showed that CD117, DOG-1, and CD34 were positive. The maximum tumor diameter in the patient G01 was 10 cm, and the mitotic index was 12/50 high-power fields (HPF). Thus, the risk in G01 was considered high. The maximum diameter of the tumor in the patient G02 was 2 cm, and the mitotic index was 1/50 HPF. Hence, the risk in G02 was defined as very low.

Tumor tissues were obtained from the resection specimens and were cut into small pieces. These pieces were digested with collagenase IV for 15 min at 37°C. Next, 70-µm cell strainer was used for filtration. Countess II Automated Cell Counter was used to determine the cell viability. Cells were loaded onto the 10X Chromium Single Cell Platform (10X Genomics) at a concentration of 1,000 cells/µL (Single Cell 3′ library and Gel Bead Kit v.3) as described in the manufacturer’s protocol. Generation of gel beads in emulsion (GEMs), barcoding, GEM-RT clean-up, complementary DNA amplification, and library construction were all performed as per the manufacturer’s protocol. Qubit was used for library quantification before pooling. The final library pool was sequenced on an Illumina NovaSeq 6000 instrument using 150-base-pair paired-end reads. Cell ranger 3.0 software was used to generate cells × genes matrixes, and all parameters were set to default. Criteria of quality control were as follows: (a) RNA counts less than 500; (b) RNA counts larger than 98% of cells; (c) mitochondrial gene expression percentage higher than 15%. A tool named CellChat was used to analyze and infer intercellular communication networks from these single-cell transcriptome data.

### Tissue microarray and immunohistochemistry

Tissue microarray with 5-micron was created from paraffin-embedded tumor tissues, which were collected from January 2011 to September 2020. The inclusion criteria were as follows: (1) all patients were diagnosed with GIST; (2) Clinicopathological information were complete. The exclusion criteria were as follows: (1) patients were treated with targeted drugs; (2) tumors were not located in stomach or intestine; (3) tissue was not used for immunohistochemistry. According to these inclusion criteria and exclusion criteria, 80 patients were included for this study. Immunohistochemistry and immunofluorescence were performed on the tissue microarray with the following antibodies: rabbit polyclonal antibodies against human CD8 (ZEN Bio, Lot No:10011860), CD206 (Protein tech, Lot No:00080496), MIF (ZEN Bio, Lot No:20200101), and CXCR4 (ZEN Bio, Lot No: BJ10221968), and monoclonal mouse antibody against human CD68 (Abcam, Lot No:00098190). ImageJ software was used to scan the stained tissue chip and count the stained cells.

### Cell lines and cell culture

Human GIST cells GIST882 with a mutation in KIT exon 13 were provided by Jonathan Fletcher (Dana-Farber Cancer Institute, Boston, MA) (19, 20). GIST882 cells were maintained in Dulbecco’s modified Eagle’s medium (DMEM, HyClone, Logan, UT) with high glucose supplemented with 10% fetal bovine serum (FBS, HyClone) and 1% penicillin/streptomycin (HyClone). GIST882 cells were identified as CD117-positive by flow cytometry.

Human THP-1 monocytes were kindly provided by Stem Cell Bank, Chinese Academy of Science. THP-1 monocytes were grown in RPMI 1640 (HyClone) supplemented with 10% FBS and 1% penicillin/streptomycin. THP-1 monocytes were treated with PMA (100 ng/mL, Sigma-Aldrich, MO, USA) to induce them to differentiate into macrophage-like cells (21).

### Detection of MIF in GIST882 cell supernatants

GIST882 cells were cultured to 60% confluence and replaced with FBS-free medium. The supernatants were collected at 24 and 48 h and stored at −80°C. The secreted MIF in GIST882 supernatants was quantified by ELISA (Beyotime Biotechnology, China) following standard protocols.

### Flow cytometric assay

Human-specific antibodies (CD206, Lot No: B318421 and CD86, Lot No: 0209406) were purchased from BD Biosciences. All samples of these groups were measured by BD flow cytometry. FlowJo software (version 7.6) (Ashland, OR, USA) was used to analyze these data.

### Real-time PCR assay

Total RNA was extracted from GIST882 cells by using TRIzol reagent (Takara Bio Inc., Japan) in accordance with the manufacturer’s instructions. Total RNA (1 μg) was reversely transcribed to form cDNA using cDNA synthesis kit (Yeasen, China). Real-time PCR was quantified using Hieff^®^ qPCR SYBR Green Master Mix Kit (Yeasen) and performed on iQ5 Opticon System (Bio-Rad, Hercules, CA). The expression of mRNA was normalized to that of glyceraldehyde-3-phosphate dehydrogenase and calculated by using the 2^−ΔΔCt^ method.

### Ethics statement

The study protocol was approved by the Ethics Committee of Sichuan Cancer Hospital (SCCHEC-02-2021-013). Written informed consent was obtained from the patients. Patient records/information were anonymized, and the methods were adopted in accordance with the approved guidelines.

### Statistical analysis

The Fisher exact test and the *t* test were used for comparison of different groups. These statistical analyses were conducted with the GraphPad Prism software version 9 and SPSS 26.0 (IBM SPSS).

## Results

### Cell composition in tumor tissue based on single-cell transcriptome analysis

**Figure 1:**
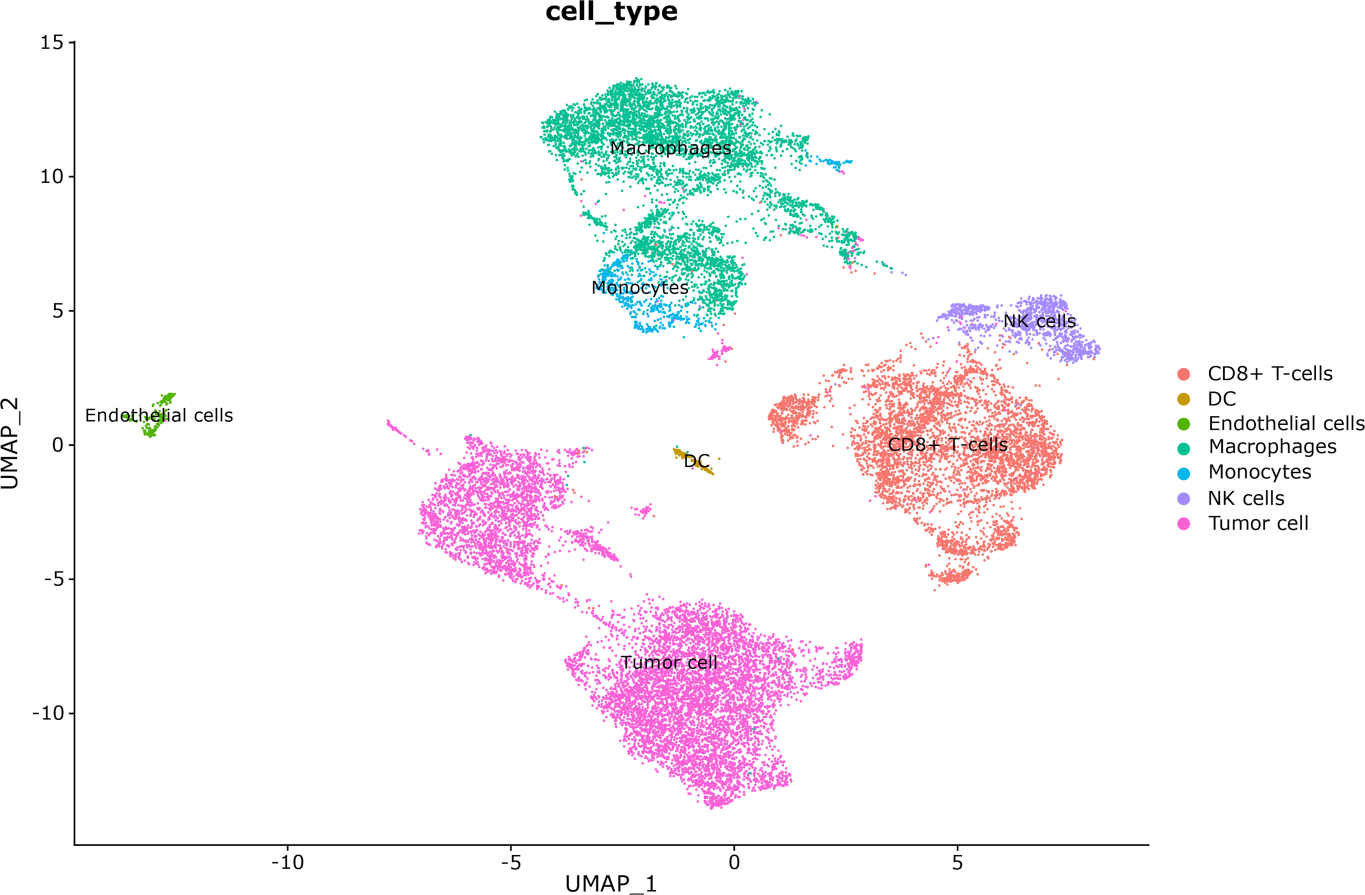
Cell clusters were visualized by uniform manifold approximation and projection (UMAP) in these two tumors.

**Figure 2:**
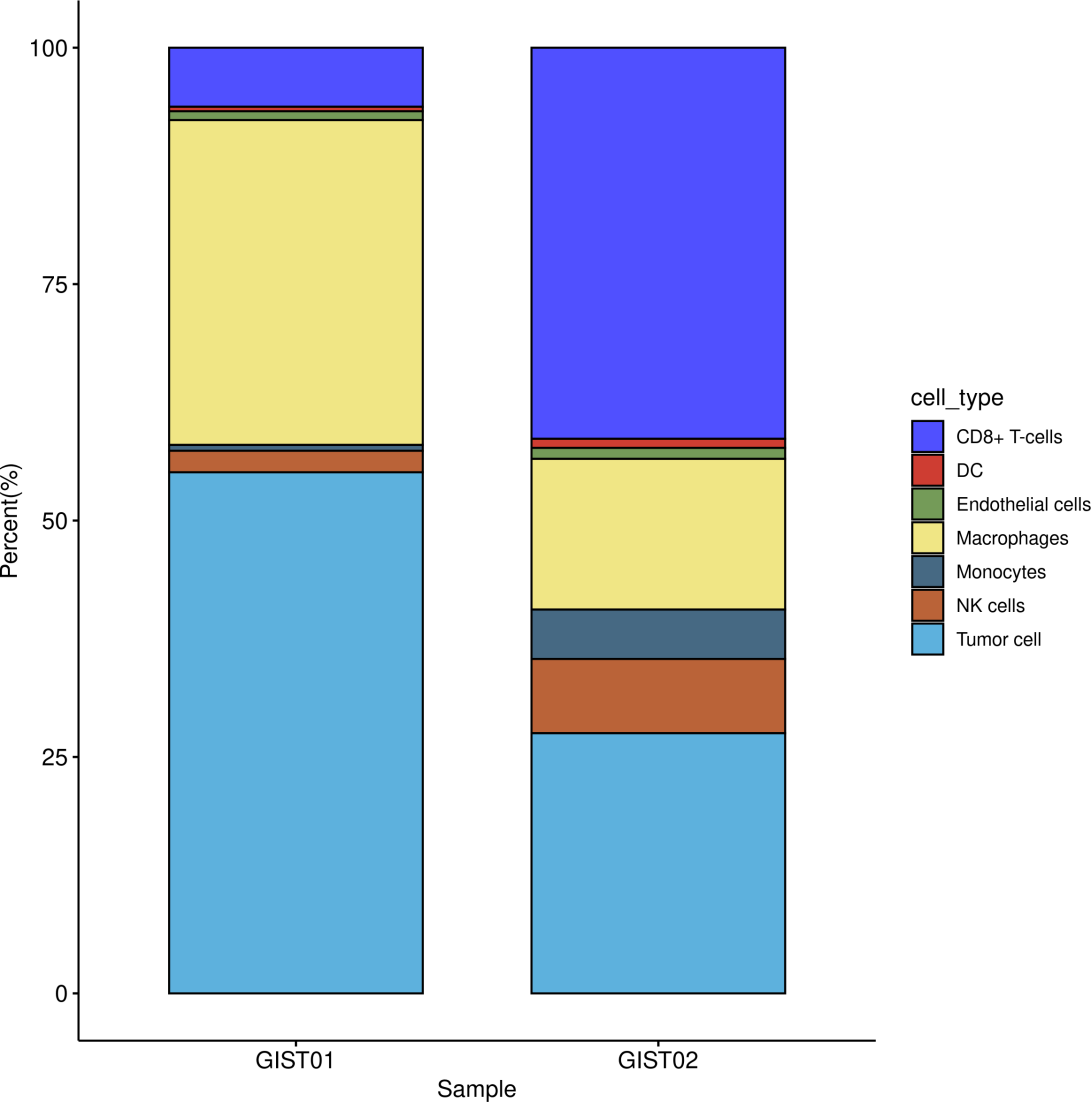
Distribution of infiltration cells in two tumors. G01: high risk GIST; G02: very low risk GIST.

In the single-cell RNA sequencing analysis, we included one patient with very-low-risk GIST and one patient with high-risk GIST. The clinicopathological characteristics are shown in **Table 1**. After quality control, 11 208 effective cells were obtained in the G01 sample (high risk), whereas 9 996 effective cells were obtained in the G02 sample (very low risk). Clusters were detected and visualized by uniform manifold approximation and projection (UMAP) (**Figure 1**). The following cell clusters were identified in the samples: tumor cells, macrophages/monocytes, CD8^+^ T cells, dendritic cells, endothelial cells, and NK cells. Immune cells were the majority of the infiltrating cells in the two tumor samples (**Figure 2**). The major cell types were the same in the two tumor samples, namely tumor cells, macrophages/monocytes, and CD8^+^ T cells. However, the counts of infiltrating cells differed between the two tumor samples (**Table 2**). In G01, the count of tumor cells was 6176, accounting for 55.1% of the total cell number; the count of macrophages was 3850, accounting for 34.3% of the total cell number; and the count of CD8^+^ T cells was 700, accounting for 6.2% of the total cell number. In G02, the count of tumor cells was 2750, accounting for 27.5% of the total cell number; the count of macrophages was 4130, accounting for 41.3% of the total cell number; and the count of CD8^+^ T cells was 1593, accounting for 15.9% of the total cell number. These results revealed that CD8^+^ T cells were more abundant in the very-low-risk GIST sample than in the high-risk GIST sample. In contrast, macrophages were less abundant in the very-low-risk GIST sample than in the high-risk GIST sample.

**Table 1:** Clinicopathological characteristics of two patients performed by single-cell RNA sequencing.

	G01	G02
Sex	male	male
Location	stomach body	stomach body
Diameter	10cm	2cm
Mitosis	12/50HPF	1/50HPF
CD34	+	+
CD117	+	+
DOG-1	+	+
Risk	high	very low

**Table 2:** Cell counts of every cluster in two samples.

Cluster	G01 (High risk)	G02 (Very low risk)
Tumor	6176 (55.1%)	2750 (27.5%)
CD8^+^T	700 (6.2%)	4134 (41.3%)
Macrophage	3850 (34.3%)	1593 (15.9%)
Monocyte	69 (0.6%)	533 (5.2%)
NK	255 (2.2%)	785 (7.8%)
DC	53 (0.4%)	96 (0.9%)
Endothelial	198 (1.8%)	116 (1.1%)

### Cell–cell communication in tumor tissue

**Figure 3:**
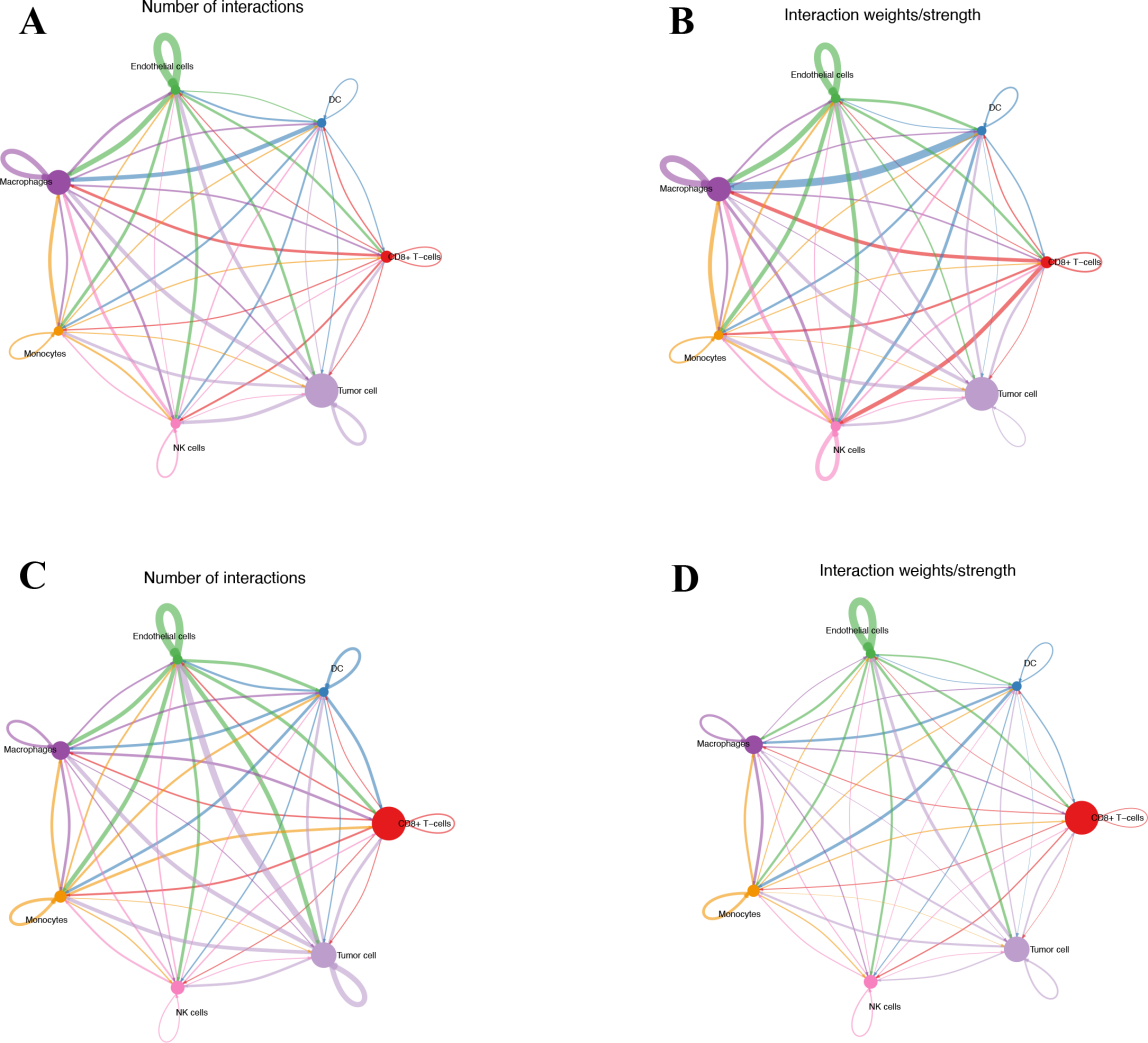
Interactions numbers and interaction weights of cell-to-cell. **(A)** Numbers of interactions in G01. **(B)** Weights of interactions in G01. **(C)** Numbers of interactions in G02. **(D)** Weights of interactions in G2. G01, high risk GIST; G02, very low risk GIST.

**Figure 4:**
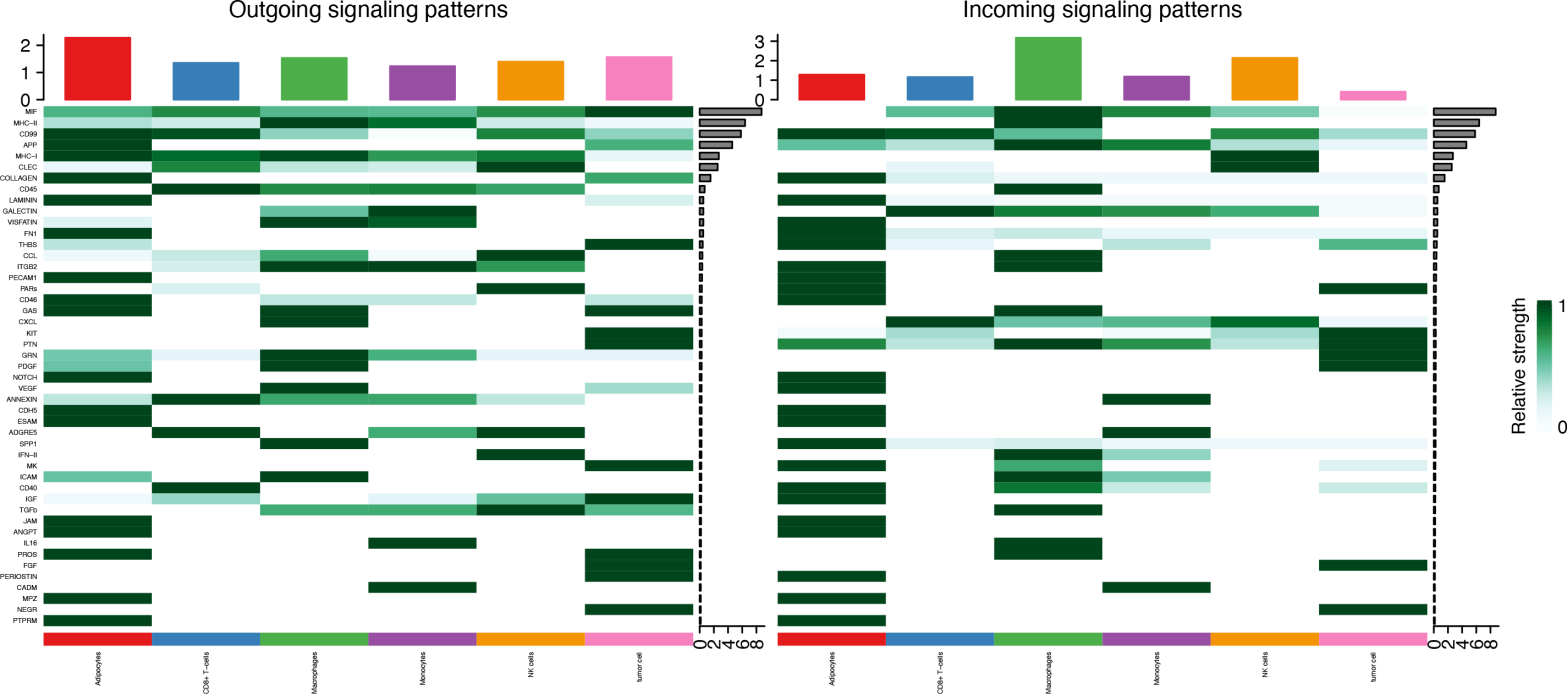
Outgoing and incoming signaling patterns of Cell-Chat in G01.

**Figure 5:**
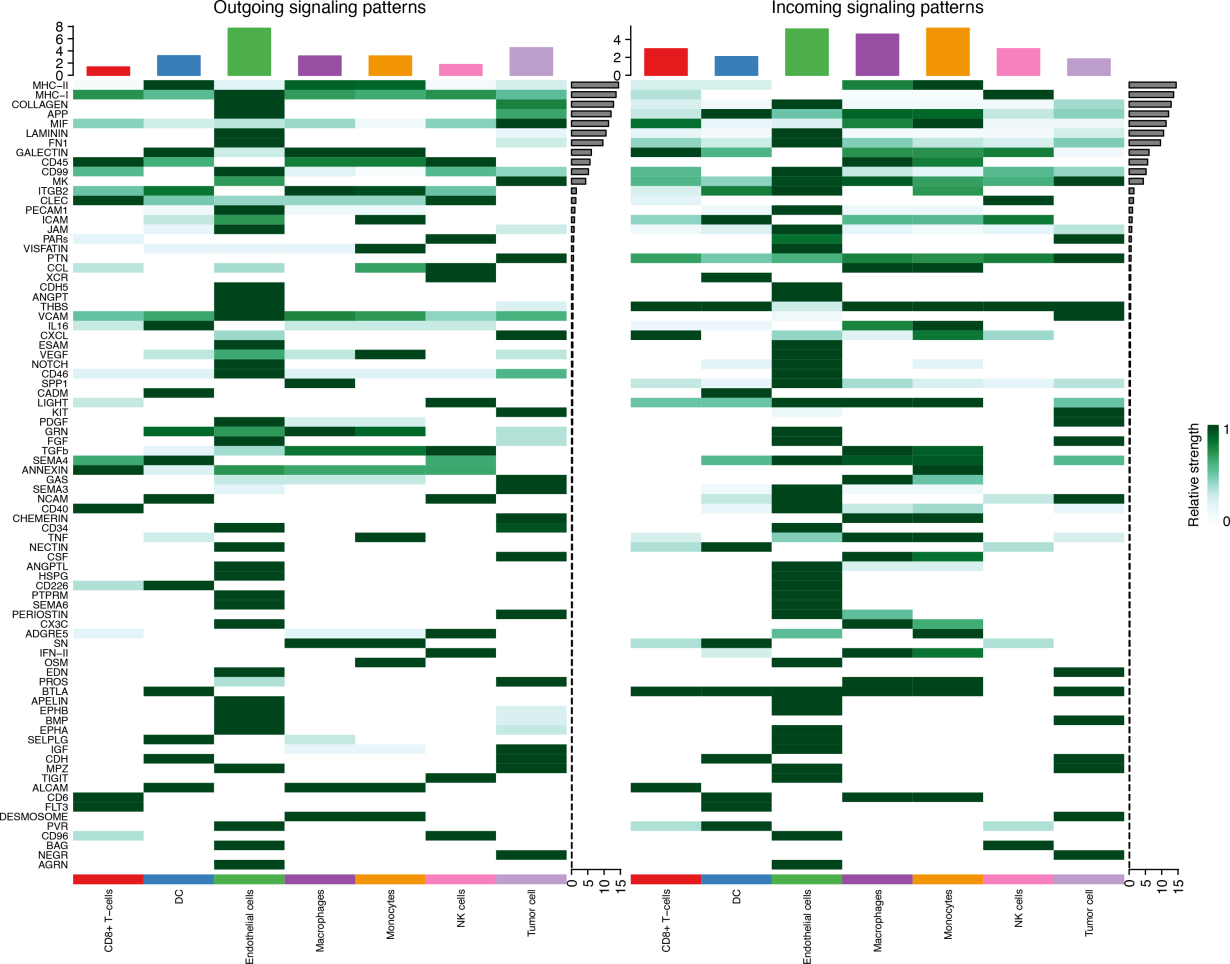
Outgoing and incoming signaling patterns of Cell-Chat in G02.

**Figure 6:**
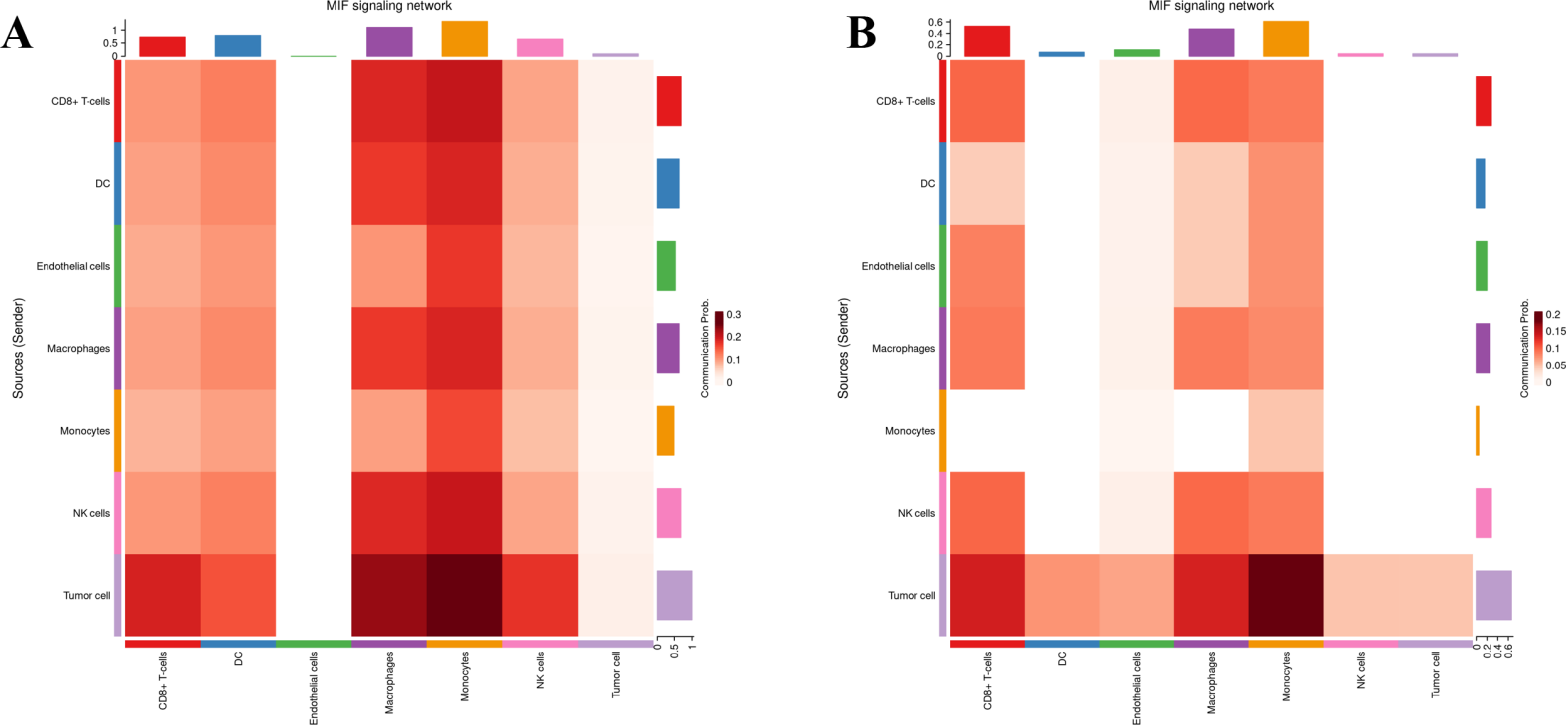
MIF/CXCR4 axis was the most crosstalk ligand-receptor between tumor cells and macrophages in G01 **(A)** and G02 **(B)**.

Seven cell types were identified in the single-cell analysis. To further show the potential interactions between these cell types, CellChat was used to quantitatively infer and analyze cell-to-cell communication networks in tumor tissues. The interaction numbers and interaction weights of cell-to-cell were analyzed in G01 and G02 as shown in **Figure 3**. The thicker lines showed more significant interaction between the cell clusters. These results showed the high interaction strength of tumor cells with macrophages and monocytes. To further investigate the significant interaction, human ligand–receptor (LR) pairs between cells were analyzed. We found that MIF/CXCR4 LR was the most common crosstalk between tumor cells and macrophages in G01 and G02 (**Figures 4**–**6**).

### Relationships of CD8, CD68, CD206, MIF, and CXCR4 expression with tumor progression as revealed by immunohistochemistry

LR interactions between tumor cells and other cells play an important role in tumor proliferation, metastasis, and progression. Thus, the MIF/CXCR4 signaling axis between tumor cells and macrophages was selected for further investigation. In this study, 80 patients were included for immunohistochemistry. Their clinicopathological characteristics are presented in **Table 3**. In these patients, 56 tumors were located in the stomach and 24 tumors were located in the intestine. Fourteen patients were classified as the very-low-risk group (LG), 31 patients were classified as the intermediate-risk group (MG), and 35 patients were classified as the high-risk group (HG).

**Table 3:** Clinicopathological characteristics of 80 patients for immunohistochemistry.

		No.
sex	male	42
	female	38
location	stomach	56
	intestine	24
diameter	≤5cm	35
	5-10cm	35
	>10cm	10
mitox	≤5	32
	5-10	21
	>10	27
risk	very low and low	14
	moderate	31
	high	35

**Figure 7:**
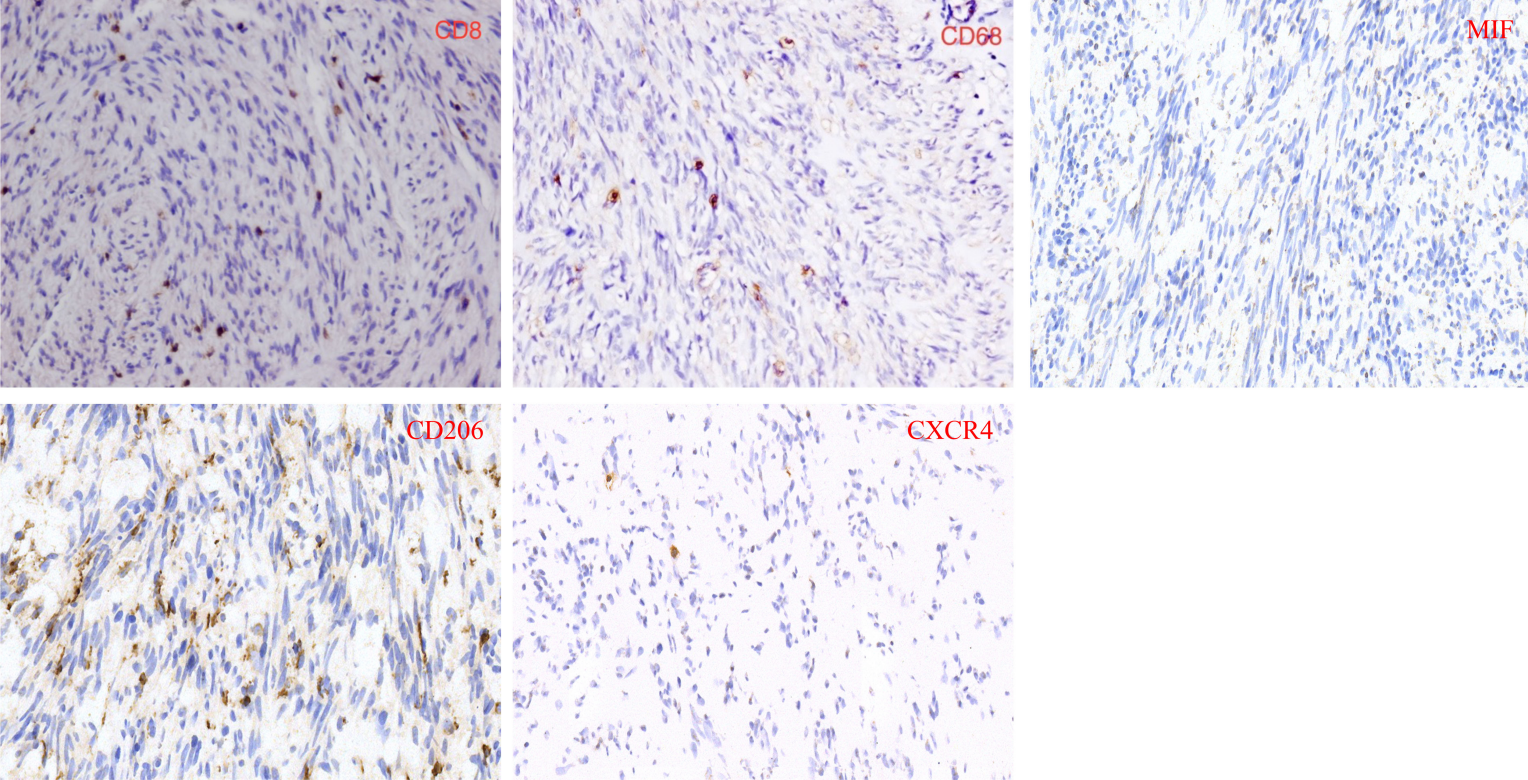
Immunohistochemical characterization of CD8, CD68, MIF, CD206 and CXCR4 in GIST samples.

**Figure 8:**
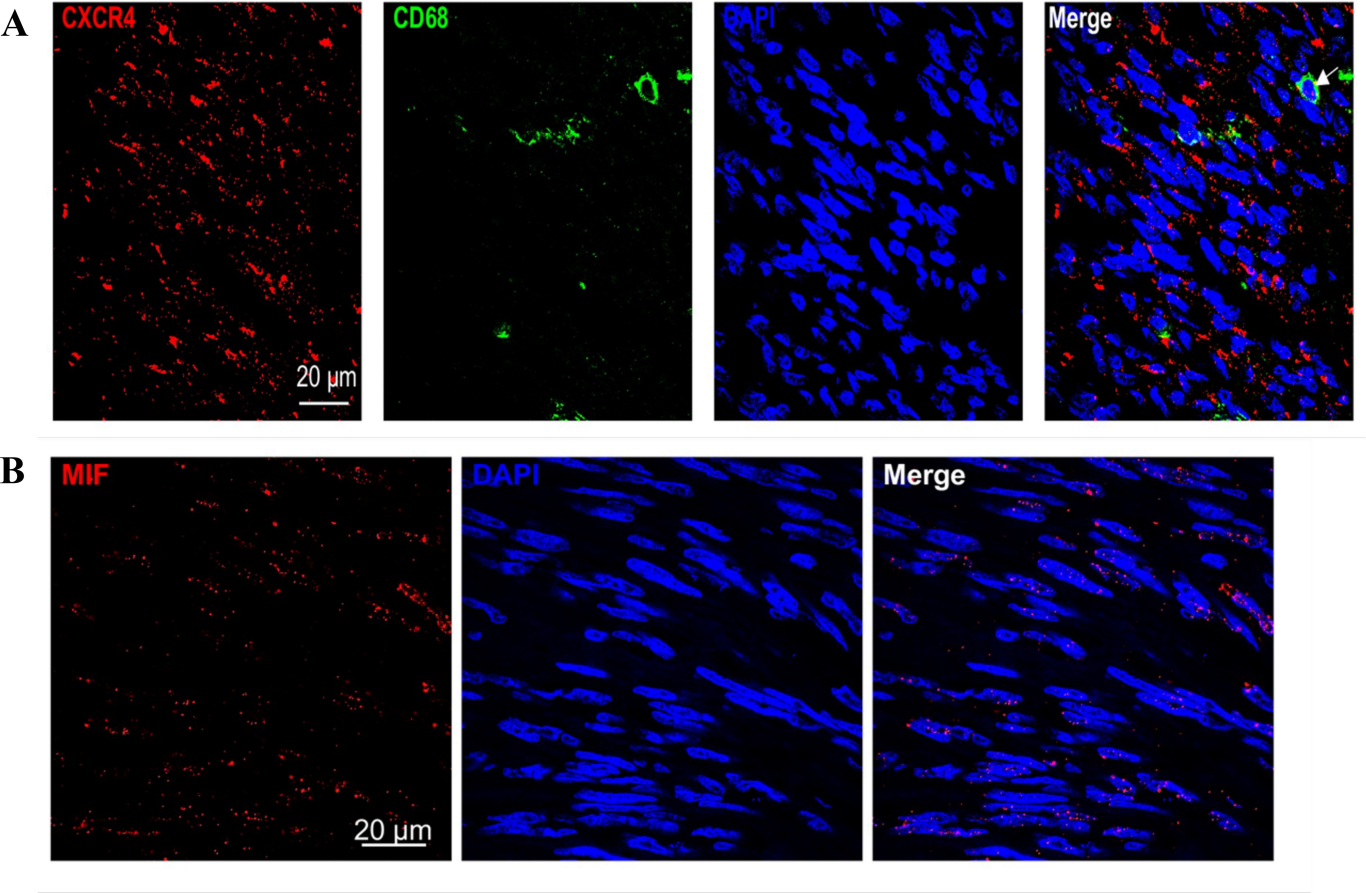
**(A)** Immunofluorescence showed that co-expression of CD68 and CXCR4 in GIST sample. **(B)** Immunofluorescence showed that MIF expression is present both inside and outside GIST cells.

**Figure 9:**
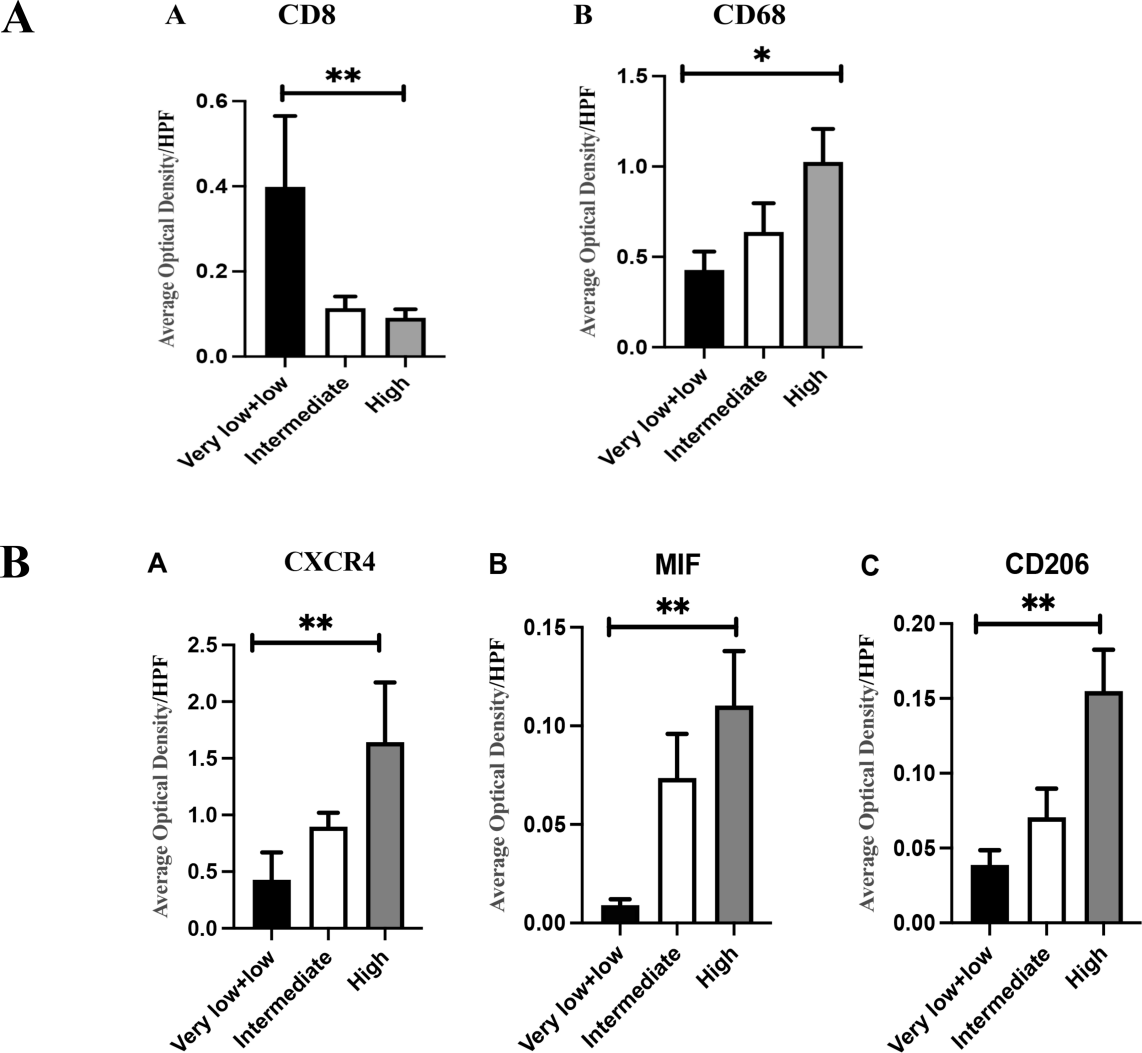
**(A)** (A) There were differences in CD8+T cell infiltration among different groups (0.35 ± 0.15 vs 0.27 ± 0.10 vs 0.09 ± 0.02, *P*=0.003); (B) There were differences in the infiltration of CD68+macrophage cells among different groups (0.38 ± 0.09 vs 0.64 ± 0.17 vs 0.98 ± 0.19, *P*=0.03). **(B)** (A) As the risk increases, the expression of CXCR4 gradually increases, and the difference is statistically significant (0.43 ± 0.24 vs 0.90 ± 0.68 vs 1.64 ± 0.53, *P*<0.001); (B) As the risk increases, the expression of MIF gradually increases, and the difference is statistically significant (0.009 ± 0.003 vs 0.70 ± 0.02 vs 0.12 ± 0.03, *P*<0.001); (C) As the risk increases, the expression of CD206 gradually increases, and the difference is statistically significant (0.04 ± 0.01 vs 0.07 ± 0.0.02 vs 0.15 ± 0.03, P<0.001); The statistical method is non-parametric test, n=80, *P<0.05; **P<0.01.

Immunohistochemistry and immunofluorescence were performed (**Figures 7**, **8**). Among the three groups, CD8+ T cells were the most abundant in the LG (LG vs. MG vs. HG: 0.35 ± 0.15 vs. 0.27 ± 0.10 vs. 0.09 ± 0.02, *P* = 0.003), whereas CD68^+^ macrophages were the most abundant in the HG (LG vs. MG vs. HG: 0.38 ± 0.09 vs. 0.64 ± 0.17 vs. 0.98 ± 0.19, *P* = 0.03) (**Figure 9A**). The expression of CD206 in the HG was the highest among the three groups (LG vs. MG vs. HG: 0.04 ± 0.01 vs. 0.07 ± 0.02 vs. 0.15 ± 0.03, *P* < 0.001). Such a phenomenon was also found in MIF expression and CXCR4 expression. Namely, among the three groups, the expression levels of MIF and CXCR4 were the highest in the HG (LG vs. MG vs. HG: 0.43 ± 0.24 vs. 0.90 ± 0.68 vs. 1.64 ± 0.53, *P* < 0.001; LG vs. MG vs. HG: 0.009 ± 0.003 vs. 0.70 ± 0.02 vs. 0.12 ± 0.03, *P* < 0.001) (**Figure 9B**). Summarily, CD8, CD68, CD206, MIF, and CXCR4 expression are related with tumor progression.

### GIST882 cells modulate M2 polarization of macrophages via MIF/CXCR4

**Figure 10:**
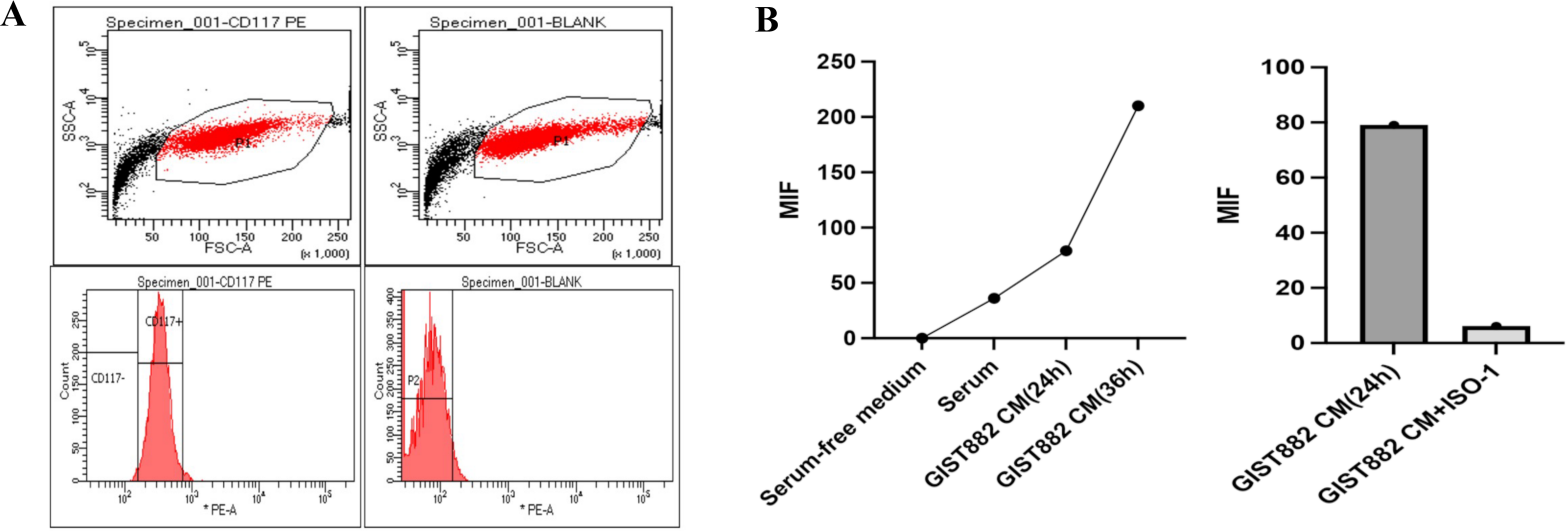
**(A)** GIST882 cells could express CD117; **(B)** GIST-882 cells could secrete MIF and MIF increased gradually along with the time. ISO-1 could inhibit the secretion of MIF.

Previous data have shown high expression of MIF in GIST tissues. To determine whether GIST-882 cell line secretes MIF, we performed an ELISA trial. As expected, GIST-882 cell line secreted MIF, and MIF gradually increased with time. When we administered ISO-1 (MIF inhibitor) to the GIST-882 cell line, secretion of MIF decreased (**Figure 10**).

**Figure 11:**
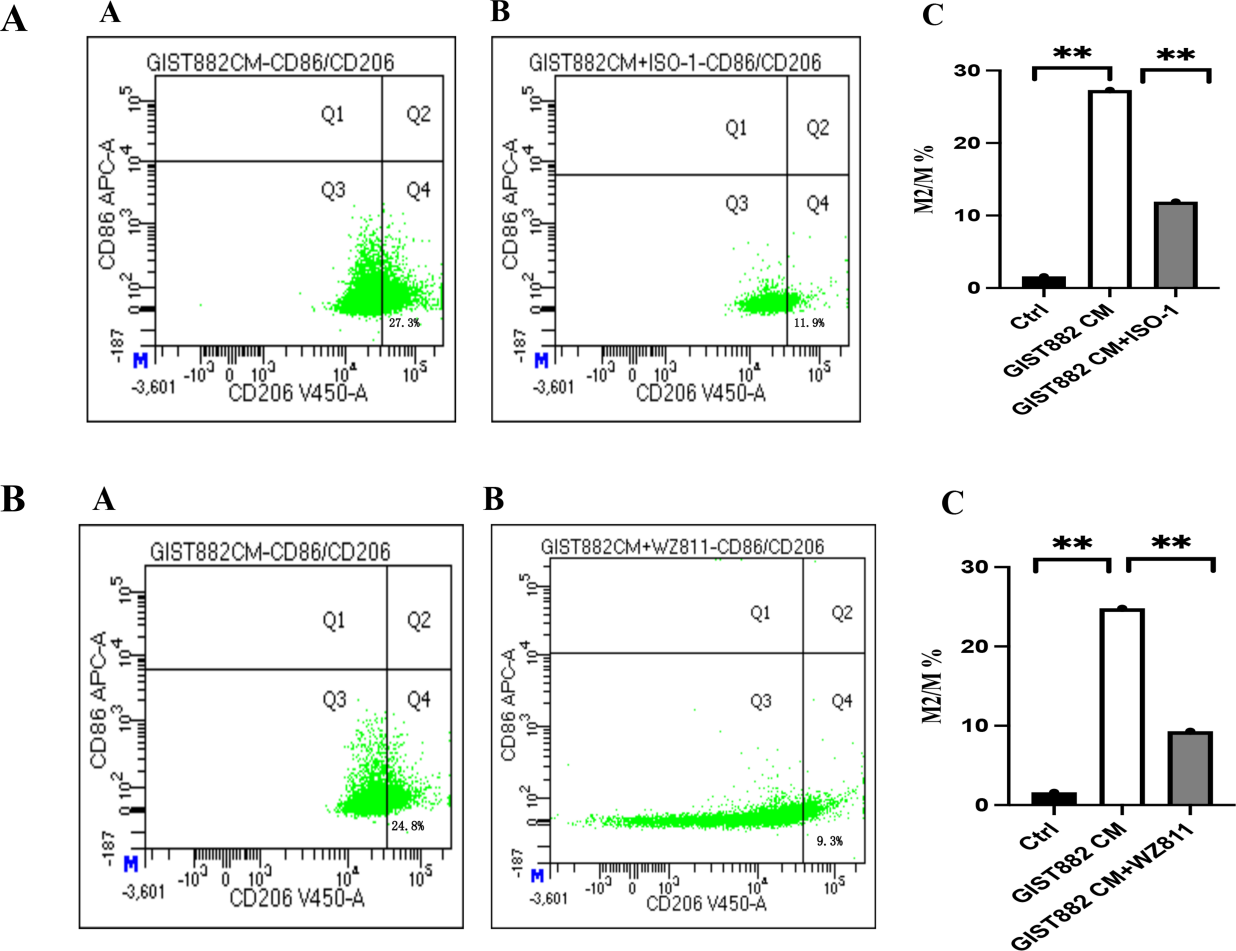
**(A)** (A) GIST882 CM group; (B) GIST882 CM+ISO-1 group; Ctrl group vs GIST882 CM group, 1.6% vs 27.3%, *P*=0.000; GIST882 CM group vs GIST882 CM+ISO-1 group,27.3% vs 11.9%, *P*=0.006; ISO-1:MIF inhibitor; The statistical method is chi square test, **:*P*<0.01. **(B)** (A) GIST882CM group; (B) GIST882CM+WZ811 group. Ctrl vs GIST882CM group:1.6% vs 24.8%, *P*=0.000; GIST882CM group vs GIST882CM+WZ811group:24.8% vs 9.3%, *P*=0.004; WZ811:CXCR4 antagonists; The statistical method is chi square test, **:*P*<0.01.

To explore the effect of GIST-882 cell line on macrophages *in vitro*, the cell supernatant was collected as conditioned medium (CM), cultured to M0 macrophages. Compared with the control group, M2 macrophages increased in the GIST882 CM group. However, when we administered ISO-1, M2 macrophages decreased. The difference between the two groups suggested that MIF was a factor to modulate M2 polarization of macrophages. Interestingly, when we administered WZ811 (CXCR4 antagonist), M2 macrophages also decreased (**Figures 11A, B**).

**Figure 12:**
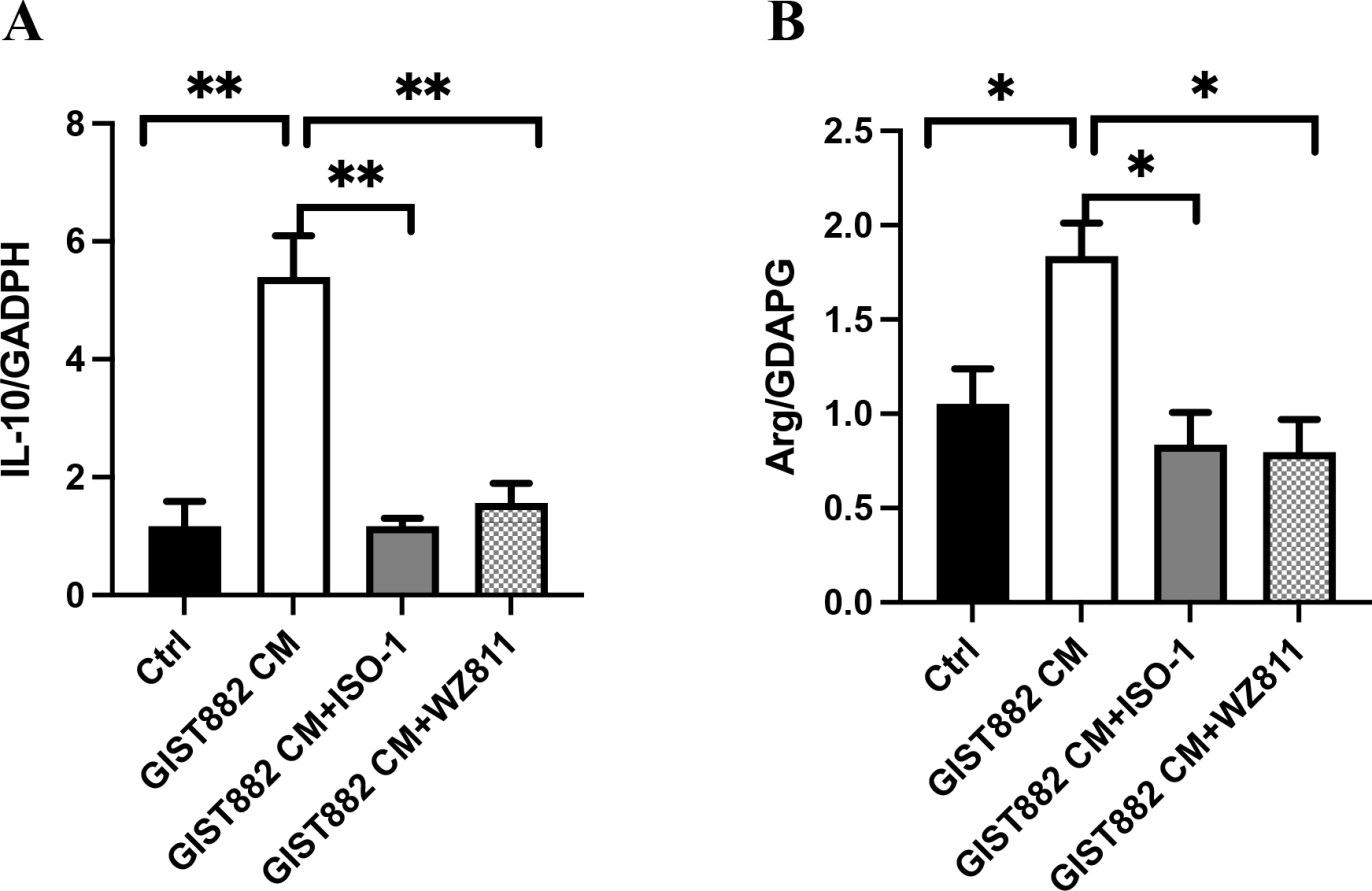
**(A)** Ctrl vs GIST882CM vs GIST882CM +ISO-1 vs GIST882CM +WZ811:1.17 ± 0.43 vs 5.40 ± 1.21 vs 1.17 ± 0.23 vs 1.57 ± 0.57; **(B)** Ctrl vs GIST882CM vs GIST882CM +ISO-1 vs GIST882CM +WZ811:1.05 ± 0.18 vs 1.83 ± 0.18 vs 0.84 ± 0.30 vs 0.80 ± 0.17; ISO-1: MIF inhibitor; WZ811: CXCR4 antagonists; The statistical method is T-test, mean ± SEM, n=3, *P<0.05; **P<0.01.

To further confirm the polarization of M2 macrophages, IL-10 mRNA and Arginase-1 mRNA were detected. The expression levels of IL-10 mRNA and Arginase-1 mRNA were in concordance with the results of flow cytometry, and were the highest in the GIST882 CM group (**Figure 12**).

## Discussion

The incidence of GIST is 1.1 per 100,000, and accounts for 80% of all gastrointestinal sarcomas (22, 23). The most common sites for GIST are the stomach (60%) and the small intestine (30%) (2, 24). Given that mutations in KIT or PDGFRA have been identified, the treatment strategy for GIST is targeted therapy. Imatinib, sunitinib, regorafenib, and ripretinib have been approved for the treatment of GIST. Imatinib, the first-line system therapy, achieves an excellent disease control in 80% of patients (25). However, drug resistance is the most challenging clinical problem. As the new era of immunotherapy has arrived, the microenvironment of GIST and the roles of infiltrating cells in tumor surveillance and tumor progression should be clarified.

CD3^+^ T cells and macrophages have been found as the most abundant tumor-infiltrating immune cells in GIST (11, 12). Our results revealed that CD8^+^ T cells and macrophages were the most abundant tumor-infiltrating immune cells. We also analyzed the differences among LG, MG, and HG. The results showed that macrophages were increased and CD8^+^ T cells were exhausted with tumor progression, which is similar to other studies (11, 18). High infiltration of macrophages predicts poor prognosis in multiple types of tumors and is considered the reason of suppressed antitumor inflammatory setting (26–28). PD-1 expression by tumor-associated macrophages has been linked to inhibition of phagocytosis and immunity (29). The M1/M2 polarization states of macrophages play an important role in tumor progression (30). The inflammatory M1 state and protumor M2 state originate from different environmental stimuli (31). However, PD-1 expression has been found in M2-state tumor-associated macrophages (29). This discovery could explain the promotion of tumor progression by M2 macrophages. To clarify the polarization of macrophages in GIST, CD206 expression was examined. We showed that CD206 expression was the highest in the HG. However, the reason for the increase in tumor-infiltrating M2 macrophages in the high-risk GIST was not clear. So, we performed a CellChat analysis to clarify the crosstalk types between tumor cells and macrophages based on single-cell RNA sequencing data (32).

Through CellChat, we quantitatively inferred and analyzed intercellular communication networks. The CellChat results showed that the MIF/CXCR4 axis was the main crosstalk type between tumor cells and macrophages. High expression of MIF has been found in many tumor tissues, such as breast cancer, lung cancer, and melanoma (33–35). It has also been revealed that high expression of MIF is closely related to tumor progression and metastasis (36). MIF could act on corresponding cells in autocrine or paracrine manner, resulting in changes in physiological functions. Currently, four receptors for MIF have been discovered, namely CD74, CXCR2, CXCR4, and CXCR7 (37–40). The MIF/CXCR4 axis has been found to contribute to cell survival, drug resistance, and tumor metastasis in multiple types of tumors (41–43).

In ELISA trial, we found that GIST882 cells were able to secrete MIF, and immunohistochemical expression of MIF and CXCR4 was related to the recurrence risk. These results suggest that the MIF/CXCR4 axis could play a key role in GIST progression. *In vitro*, we found that the MIF/CXCR4 axis contributed to M2 polarization of macrophages. The expression levels of MIF and CXCR4 have been identified as adverse prognostic factors in multiple types of tumors (43–45). The MIF/CXCR4 axis could contribute to drug resistance in tumor invasion and metastasis (41, 43). However, the mechanism of the MIF/CXCR4 axis in this process is unclear. Our results provide an idea to explain this phenomenon and stimulate further research.

In conclusion, macrophages are the most abundant infiltrating cells in GIST. The MIF/CXCR4 axis is the most ligand–receptor interaction between macrophages and tumor cells. GIST cells can regulate macrophage M2 polarization through the MIF/CXCR4 axis to form an immunosuppressive microenvironment.

## Data Availability

The names of the repository/repositories and accession number(s) can be found the China National Genebank (CNGB, https://db.cngb.org/cnsa/). The number of these data was sub062300 (sub062501, CSE0000443).
